# Drug discovery using clinical outcome-based Connectivity Mapping: application to ovarian cancer

**DOI:** 10.1186/s12864-016-3149-5

**Published:** 2016-10-19

**Authors:** Rama Raghavan, Stephen Hyter, Harsh B. Pathak, Andrew K. Godwin, Gottfried Konecny, Chen Wang, Ellen L. Goode, Brooke L. Fridley

**Affiliations:** 1 Department of Biostatistics, University of Kansas Medical Center, 3901 Rainbow Blvd, Kansas City, KS 66160 USA; 2 Department of Pathology and Laboratory Medicine, University of Kansas Medical Center, Kansas City, KS 66160 USA; 3 Department of Medicine, Hematology & Oncology, University of California – Los Angeles, Los Angeles, CA 90095 USA; 4 Department of Health Sciences Research, Mayo Clinic, Rochester, MN 55901 USA

**Keywords:** Gene expression signature, Time to recurrence, Bioinformatics, Connectivity Mapping, Drug discovery, Ovarian cancer

## Abstract

**Background:**

Epithelial ovarian cancer (EOC) is the fifth leading cause of cancer death among women in the United States (5 % of cancer deaths). The standard treatment for patients with advanced EOC is initial debulking surgery followed by carboplatin-paclitaxel combination chemotherapy. Unfortunately, with chemotherapy most patients relapse and die resulting in a five-year overall survival around 45 %. Thus, finding novel therapeutics for treating EOC is essential. Connectivity Mapping (CMAP) has been used widely in cancer drug discovery and generally has relied on cancer cell line gene expression and drug phenotype data. Therefore, we took a CMAP approach based on tumor information and clinical endpoints from high grade serous EOC patients.

**Methods:**

We determined tumor gene expression signatures (e.g., sets of genes) associated with time to recurrence (with and without adjustment for additional clinical covariates) among patients within TCGA (*n* = 407) and, separately, from the Mayo Clinic (*n* = 326). Each gene signature was inputted into CMAP software (Broad Institute) to determine a set of drugs for which our signature “matches” the “reference” signature, and drugs that overlapped between the CMAP analyses and the two studies were carried forward for validation studies involving drug screens on a set of 10 EOC cell lines.

**Results:**

Of the 11 drugs carried forward, five (mitoxantrone, podophyllotoxin, wortmannin, doxorubicin, and 17-AAG) were known a priori to be cytotoxics and were indeed shown to effect EOC cell viability.

**Conclusions:**

Future research is needed to investigate the use of these CMAP and similar analyses for determining combination therapies that might work synergistically to kill cancer cells and to apply this *in silico* bioinformatics approach using clinical outcomes to other cancer drug screening studies.

**Electronic supplementary material:**

The online version of this article (doi:10.1186/s12864-016-3149-5) contains supplementary material, which is available to authorized users.

## Background

The American Cancer Society estimates that in 2015, approximately 14,180 women will die of epithelial ovarian cancer (EOC) in the United States, with 21,290 estimated new cases [1]. Since early ovarian cancer shows few symptoms, the vast majority of patients continue to be diagnosed with advanced stage disease, where the prognosis is poor with 5-year survival rate around 27 %. The standard treatment protocol for patients with advanced EOC is an initial debulking surgery, followed by carboplatin-paclitaxel combination chemotherapy. Platinating agents, such as cisplatin, carboplatin, and oxaliplatin, are able to interact with DNA to form monoadducts, intra- and interstrand crosslinks, and DNA-protein crosslinks, ultimately resulting in cell death. Taxane agents are widely used chemotherapeutic drugs often in combination with platinating agents. Taxanes, such as paclitaxel and docetaxel, block cell division by binding to β-tubulin, stabilizing the microtubules, leading to cell death [2, 3]. Although over 70 % of ovarian cancer patients are initially sensitive to the combination therapy consisting of a platinum agent and a taxane, the majority will experience relapse and subsequent resistance to the therapy [4]. Thus, finding new therapeutic options for treating patients with EOC is essential.

One approach that has been used widely in cancer drug discovery is Connectivity Mapping [5]. The Connectivity Map (also known as “CMAP”) is a collection of genome-wide transcriptional expression data from cultured human cells treated with bioactive small molecules analyzed using pattern-matching algorithms that discover relationships between the drugs, gene expression changes, and the phenotypes. This computational approach has greatly facilitated drug screening studies, as CMAP contains more than 7000 gene expression profiles for approximately 1300 compounds (https://www.broadinstitute.org/cmap/). In particular, it has been employed in many studies for discovering repurposing drugs against common diseases, including diabetes and Alzheimer’s disease [5], and for treating solid tumors, including those associated with colon cancer [6], breast cancer [7], and lung adenocarcinoma [8]. The basic approach for CMAP-based drug discovery studies is the identification of disease- (or phenotype) associated genomic signatures that inversely correlate with perturbation in the genomic signature associated with the administration of molecules or drugs [5]. In these studies, the essence of the protocol – the individual-gene CMAP approach – for identifying drugs for treating a specific disease is straightforward: find a set of differentially expressed genes (DEGs) obtained by comparing two sets – e.g., control and patient tissues – of gene expression microarrays, score the match between the DEG set and genomic profiles of drugs given by CMAP, and rank the drugs by score [9, 10]. The candidate drugs are those with the highest absolute scores.

However, these studies have some limitations. First, the list of DEGs used in the CMAP analysis is usually based on a relatively small number of biological replicates (i.e., a handful of cancer cell lines). Additionally, the number of cell lines in CMAP exposed to the compounds is limited to only a handful of cancer cell lines from breast, leukemia, prostate and melanoma, with each compound usually tested on only a few cell lines. Furthermore, recent studies have shown potential issues with use of cancer cell lines in terms of the lack of rigor in the estimation of drug response phenotypes in cell lines [11] and the lack of concordance between cell lines and human genomic profiles [12]. Among EOC cell lines, a recent study has found that IGROV1 is most probably not of the high grade serous subtype as it is often quoted [13]. To address the limitations of previous DEG selection based on cancer cell lines, we determined our DEGs based on two large collections of tumor gene expression data collected on high grade serous EOC patients for whom clinical endpoints were available (407 and 326 cases in TGCA and Mayo Clinic studies, respectively). To determine the most relevant DEGs, we characterized the associations between gene expression and time to recurrence. We hypothesized that the potential therapeutic drugs for EOC are those that have a gene expression profile that are related to the gene expression signature related to clinical outcome of time to recurrence (TTR). Following CMAP analyses, we then tested key genes on ten EOC cell lines to assess the ability of the candidate drugs to effectively kill EOC cells.

## Methods

### TCGA ovarian cancer study

As part of TCGA, research collected and assessed genome-wide gene expression data on 518 samples using the Agilent Expression 244 K microarray. Gene expression and clinical data were downloaded from the TCGA Data Portal (https://tcga-data.nci.nih.gov/tcga/dataAccessMatrix.htm) on September 17, 2012. Gene expression data were lowess normalized with replicate probes for a gene collapsed by averaging across the probes. Of 518 serous cyst adenocarcinoma with Agilent gene expression data, 449 of the tumors were classified as high-grade. Thus, restricting to high grade serous tumors and removing the samples in the TCGA that were part of the Mayo Clinic study, we included 407 tumors in our analysis. Summary of the TCGA participants are presented in Table 1.

**Table 1:** Summary of TCGA and Mayo Clinic Studies

	TCGA (*n* = 407)	Mayo Clinic (*N* = 326)
Time to recurrence/Progression free survival		
Median TTR	15.3 (months)	13.0 (months)
No recurrence/No progression	49.87 (%)	31.59 (%)
Recurrence/Progression	48.40 (%)	67.79 (%)
Stage		
I	3.19 (%)	1.53 (%)
II	4.17 (%)	3.06 (%)
III or IV	92.13 (%)	95.39 (%)
Age		
Mean, [Q1, median, Q3]	59.8 [53, 60, 68]	58.3 [50, 55.5, 66.25]
Surgical debulking		
Optimal	65.11 (%)	73.92 (%)
Sub-optimal	23.83 (%)	24.84 (%)

### Mayo clinic ovarian cancer study

Briefly, eligible cases were ascertained between 1992 and 2009 at the Mayo Clinic within 1 year of diagnosis with pathologically confirmed primary invasive high-grade serous EOC. Progression and vital status were obtained from the Mayo Clinic Tumor Registry, electronic medical records, and active patient contact. All cases provided written informed consent for use of their tissues and medical records in research; all protocols were approved by the Mayo Clinic Institutional Review Board. RNA from fresh frozen tumors of each patient was extracted and assessed using Agilent Whole Human Genome 4 × 44 K Expression Arrays as previously described [14, 15]. The program “ComBat” was used to correct for batch-effects due to Cy5 and Cy3 labeling differences observed among experimental batches [16]. Summary of 326 Mayo Clinic participants are presented in Table 1. Data used in this study can be found at the Gene Expression Omibus (GSE73614, GSE53963 and GSE74357).

### Statistical and CMAP analyses

Cox proportional hazard models were used to assess the association of gene expression (gene-by-gene) with time to recurrence (TTR), by study with and without adjustment for age at diagnosis, stage, and debulking status. DEG probes were selected for inclusion in the CMAP analysis if *p* < 0.01 for the unadjusted analyses and *p* < 0.005 for the covariate adjusted analyses; different thresholds were used as CMAP (Broad Institute) has a limit on the number of features included in any signature. These set of probes were then mapped to genes and then to the Affymetrix ID, as CMAP is based on Affymetrix probes/features. We conducted CMAP analysis on individual genes with hazard ratio (HR) > 1 coded as “positively” associated and genes with HR < 1 coded as “negatively” associated with TTR to determine a set of drugs for which our gene signatures matches the “reference” signature (either positively or negatively). Clustering of samples based on gene expression levels was completed using recursive partition mixture models using the R package *RPMM* [17], restricting the number of levels to a maximum of 2.

### In vitro drug screens

In vitro drug cytotoxicity assays were conducted to determine which of the drugs highlighted by CMAP analysis affected viability of EOC cells. The drugs were purchased from the following vendors: cotinine, 3-nitropropionic acid, adiphenine hydrochloride, ethosuximide, and podophyllotoxin (Sigma); cephalexin and mitoxantrone (Selleckchem); clemizole (BioVision); wortmannin, doxorubicin, and 17-AAG (LC Laboratories). Upon receipt, dimethyl sulfoxide (DMSO) was used to prepare 10 mM stock solutions for all of the drugs except for cephalexin, which was prepared at a 5 mM concentration due to reduced solubility. Single-use aliquots of the stock drug solutions were made and stored at −80 °C.

All cell lines used in this study were obtained or derived at the Fox Chase Cancer Center (Philadelphia, PA). Details of the origin of the EOC cell lines (*N* = 10: A1847, A2780, C30, CP70, OVCAR4, OVCAR5, OVCAR8, OVCAR10, PEO4, and SKOV3) have been previously reported [18–20]. Each cell line was grown in RPMI 1640 (Corning Cellgro) containing 2 mM L-glutamine and supplemented with 10 % FBS (Gibco), 100 I.U./mL penicillin (Corning Cellgro), 100 μg/mL streptomycin (Corning Cellgro), and 7.5 μg/mL insulin (Gibco) and maintained at 37 °C in a humidified atmosphere with 5 % CO2. Cell lines were grown to 80 % confluency, harvested and seeded into 96-well plates at concentrations of 2000 to 4000 cells per well in a total volume of 95 μL. Twenty-four hours after seeding, drug compounds were prepared using cell growth media and 5 μL of each were added to the seeded cells in the 96-well plates. A Microlab Nimbus 96 pipetting robot (Hamilton) was used to prepare the serial dilutions and for addition to the cell lines. The final drug solutions consisted of eight concentrations ranging from 20 to 0.0012 μM (serial four-fold dilutions). Vehicle-only wells were included on each plate to serve as interplate normalization controls.

Seventy-two hours following drug addition, a 1/5th volume of CellTiter Blue reagent (Promega) was added directly to each well using a Matrix WellMate (Thermo Scientific). The plates were incubated at 37 °C for 150 min and the fluorescent signal was measured using an Infinite® M200 Pro microplate reader (Tecan). The ratio of the fluorescent signal in a drug treated well to that of the average fluorescent signal from the vehicle treated wells on each plate multiplied by 100 was used to calculate cell viability for each drug treated well for each cell line. A minimum of two biological replicates were performed for each cell line. The viability data were subjected to non-linear regression analysis and IC_50_ values calculated using Prism 5 software (GraphPad). All data in the viability curves are reported as mean ± standard error of the mean (SEM).

## Results

### Genes associated with clinical outcome

To determine the gene signatures to use in CMAP analyses, we determined the set of probes (and corresponding gene) associated with TTR by fitting separate Cox proportional hazards models within each study (*N* = 407 for TCGA, *N* = 326 for Mayo Clinic) with and without adjustment for age at diagnosis, stage, and debulking status. Comparing the genes associated with TTR with no adjustment for covariates at the 0.05 significance level and the same direction of effect resulted in 143 genes in common between TCGA and Mayo studies; 96 genes had a hazard ratio (HR) < 1 (high expression associated with better outcome) and 47 genes had a HR > 1 (high expression associated with worse outcome). In contrast, the adjusted analyses resulted in 186 genes in common (*p* < 0.05, HR in same direction), with 128 genes having a HR < 1 and 58 having a HR > 1. Table 2 presents the genes with *p* < 0.01 in both studies (with same direction of effect) for both the adjusted and unadjusted analyses.

**Table 2:** Genes associated with epithelial ovarian cancer time to recurrence in analysis of tumors from the TCGA and Mayo Clinic (*p* < 0.01) for both adjusted and unadjusted analyses

Analysis	Gene	TCGA study			Mayo Clinic study		
		HR	95 % CI	P	HR	95 % CI	P
Adjusted for covariates	*PTPRCAP*	0.71	(0.56,0.89)	0.004	0.14	(0.04,0.40)	3.6E-04
	*UBASH3A*	0.78	(0.65,0.92)	0.005	0.19	(0.06,0.50)	0.001
	*PPBP*	1.18	(1.04,1.33)	0.009	5.79	(1.94,17.21)	0.002
	*PVRIG*	0.73	(0.58,0.91)	0.005	0.28	(0.12,0.62)	0.002
	*IGKV3-20*	0.85	(0.76,0.93)	0.001	0.41	(0.23,0.72)	0.002
	*FCRL5*	0.84	(0.74,0.95)	0.008	0.30	(0.14,0.65)	0.002
	*ITK*	0.83	(0.72,0.95)	0.008	0.30	(0.13,0.66)	0.003
	*IGHV3OR16-12*	0.85	(0.77,0.94)	0.002	0.47	(0.28,0.78)	0.004
	*VANGL1*	0.67	(0.51,0.88)	0.004	0.23	(0.08,0.62)	0.004
	*SLC16A8*	1.23	(1.06,1.43)	0.006	4.55	(1.58,13.01)	0.005
	*IGKV3-20*	0.89	(0.82,0.96)	0.004	0.50	(0.30,0.83)	0.007
	*ACAP1*	0.78	(0.65,0.93)	0.006	0.23	(0.07,0.68)	0.008
Unadjusted for covariates	*VANGL1*	0.73	(0.58,0.92)	0.008	0.26	(0.09,0.69)	0.007
	*CD38*	0.88	(0.80,0.96)	0.007	0.47	(0.28,0.76)	0.003
	*ELA1*	1.42	(1.10,1.82)	0.006	4.93	(1.97,12.32)	0.001
	*PSME2*	0.76	(0.62,0.91)	0.004	0.16	(0.04,0.53)	0.003
	*UBD*	0.92	(0.87,0.97)	0.004	0.59	(0.43,0.81)	0.001

Note, three Agilent probes did not have a current HUGO gene ID and are not presented in the table

To further evaluate that these sets of genes were predictive of outcome, we clustered the samples based on the expression levels of the genes in common between the two studies using recursive partitioning mixture models (RPMM). TCGA and Mayo Clinic samples were clustered separately on the set of genes in common between the two studies (with and without adjusting for covariates). The resulting cluster assignments were then assessed for association with survival using log-rank tests. The log-rank p-values for testing cluster assignment with TTR were 0.012 and 0.015 for TCGA and Mayo Clinic studies (genes in common with no adjustment for covariates) (Additional file 1: Figure S1) and 0.016 and 0.112 based on clustering of genes from model adjusting for covariates (Additional file 2: Figure S2).

### Connectivity Mapping

**Fig. 1:**
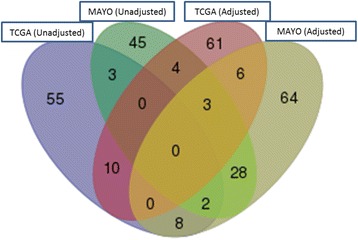
Venn diagram depicting the overlap of compounds/drugs found from the CMAP analyses

CMAP analysis was completed for each of the 4 DEG sets (TCGA/Unadjusted Analyses; TCGA/Adjusted Analyses; Mayo/Unadjusted Analyses; Mayo/Adjusted Analyses) using the CMAP software developed at the Broad Institute (https://www.broadinstitute.org/cmap/). This resulted in (*p* < 0.05): 78, 84, 85 and 111 drugs signatures were found to be either negatively or positively related with gene signatures based on analysis of TCGA (unadjusted), TCGA (adjusted), Mayo Clinic (unadjusted) and Mayo Clinic (adjusted), respectively. We then looked at the overlap of the compounds found in both studies and found that 9 (and 5) compounds were in common between CMAP analyses based on the covariate adjusted (unadjusted) signature, as illustrated in Fig. 1. This set of compounds included the following: tanespimycin (17-AAG), ethosuxiumide, cotinine, clemizole, 0175029–0000 (unadjusted for covariates); wortmannin, 3-nitropropionic acid, adiphenine, cephaeline, doxorubicin, podophyllotoxin, mitoxantrone, cephalexin, 5182598 (adjusted for covariates). In vitro drug screens were completed using these drugs with the exception of 0175029–0000 and 5182598 for which commercial sources were not found and cephaeline which was cost prohibitive.

### In vitro drug screens

**Fig. 2:**
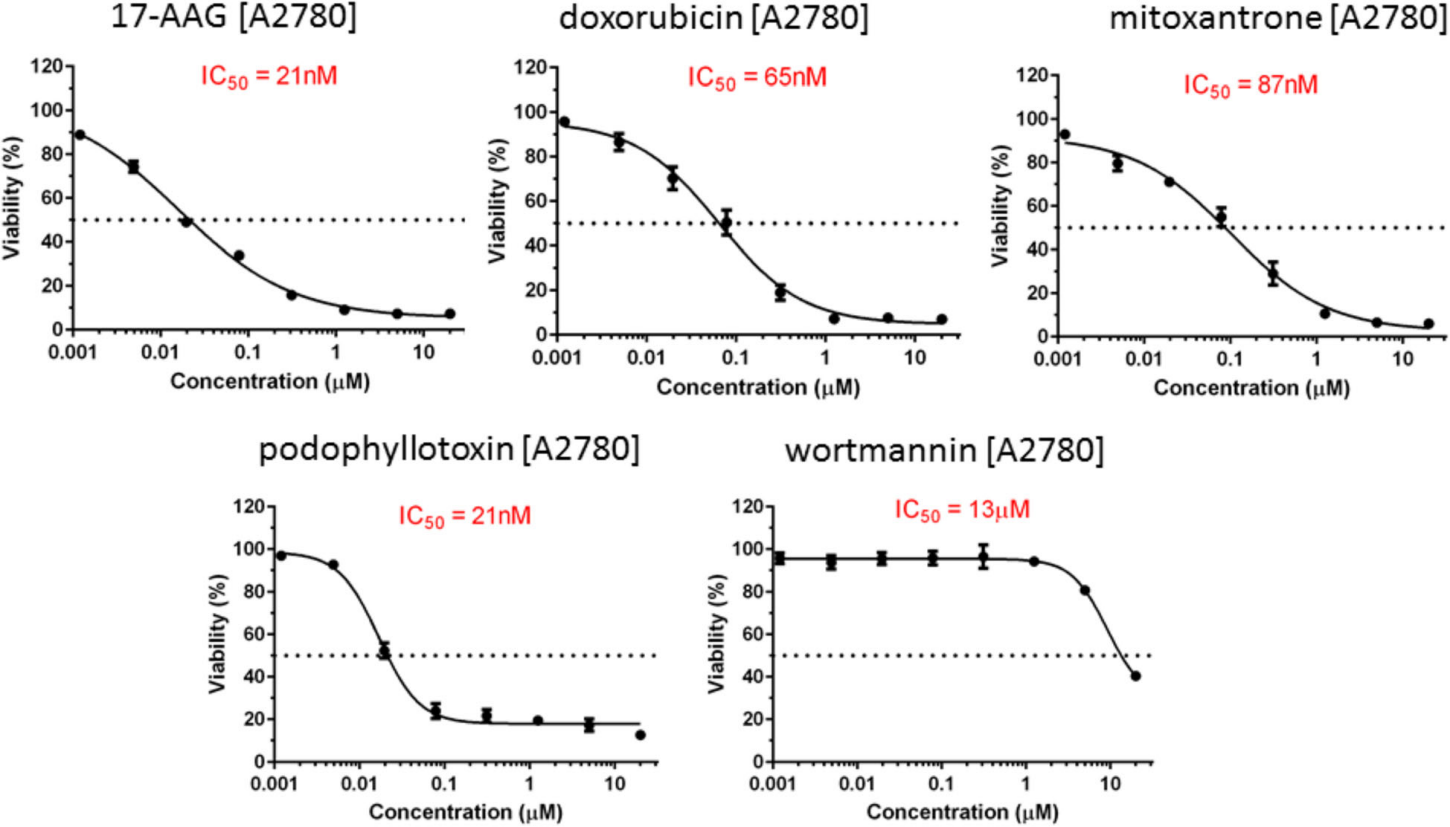
Drug dose response curves for five compounds for ovarian cancer cell line A2780: mitoxantrone, podophyllotoxin, wortmannin, doxorubicin, and 17-AAG

In order to experimentally determine the effect of the eleven CMAP-highlighted drugs on EOC cell viability, we conducted in vitro dose response studies on a set of 10 EOC cell lines. A significant reduction in cell viability following 72 h of drug treatment was observed for five of these compounds: mitoxantrone, podophyllotoxin, wortmannin, doxorubicin, and 17-AAG (Fig. 2). These 5 drugs were known to be cytotoxic and therefore were expected to affect EOC cell growth and viability based on their mechanisms of action [21–28]. The six remaining drugs failed to show in vitro anti-cancer activity, consistent with prior reports of minimal cytotoxic capacity [29–34]. The dose response data for all 11 drugs across the 10 EOC cell lines can be found in Additional file 3: Figure S3, Additional file 4: Figure S4, Additional file 5: Figure S5, Additional file 6: Figure S6, Additional file 7: Figure S7, Additional file 8: Figure S8, Additional file 9: Figure S9, Additional file 10: Figure S10, Additional file 11: Figure S11, Additional file 12: Figure S12 and Additional file 13: Figure S13.

## Discussion

In this manuscript we have presented a bioinformatics approach for connectivity mapping based on clinical outcomes collected on a large patient population followed by functional validation of the identified drugs. The strengths of this approach are: non-reliance on a signature determined based on a small number of cancer cell lines; use clinically relevant outcomes that are directly tied to response (i.e., time to recurrence or overall survival); large clinical studies that provide the ability to look at overlap between CMAP determined drugs; functional validation to confirm the ability of the discovered drugs to kill ovarian cancer cells. The choice of applying this approach to ovarian cancer was by design, as EOC treatment is somewhat uniform where the majority of patients undergo tumor debulking or cytoreduction surgery to remove as much of the tumor as possible, followed by platinum-taxane combination chemotherapy. Application of this approach to other cancers with less standard treatments will introduce heterogeneity into clinical outcome and would need to be accounted for in the statistical analyses (if possible). Nevertheless, there are still limitations to CMAP analysis, which include: small sample size contributing to the CMAP database, with none of the cell lines having been derived from EOC; “batch effects” in CMAP cell line cultures [35]; and the signatures are based on only gene expression measured using microarrays.

This study is a proof of principle that clinical outcomes from large studies (of which one is publically available for research) have the ability to be leveraged for drug discovery. Of the 11 drugs carried forward, we a priori hypothesized that 5 of these drugs would affect EOC cell viability (mitoxantrone, podophyllotoxin, wortmannin, doxorubicin, and 17-AAG), for which all 5 showed an ability to kill EOC cells in vitro (cell lines were treated using serial dilutions of the drugs (0 to 20 μM) for 72 h followed by measuring cell viability using the CellTiter-Blue assay). Their anti-cancer activities have been studied extensively and mitoxantrone and doxorubicin have been used in the treatment of a variety of cancers [36–43]. However, the CMAP analyses of the TCGA and Mayo Clinic studies did not identify the two most commonly used therapies for EOC, with none of the 11 drugs identified having similar structure or mechanism of action to carboplatin or paclitaxel. However, three of the 11 compounds are topoisomerase II inhibitors (doxorubicin, podophyllotoxin, mitoxantrone), a class of drugs used often in the treatment of breast cancer, lung cancer, testicular cancer, lymphomas and sarcomas [44].

The natural products podophyllotoxin, wortmannin, and 17-AAG have proven highly toxic to human subjects but subsequent analogues such as etoposide, PX-866, and ganetespib, respectively, have decreased side effects and are currently being investigated in the treatment of EOC [13, 45, 46]. The six compounds that failed to exhibit significant in vitro cytotoxicity across the EOC cell line panel belong to classes of drugs related to central nervous system pathways, exhibit antimicrobial activity or are required in high micromolar concentrations which may not be physiologically achievable. Interestingly, adiphenine was also identified using CMAP analysis as an adjuvant therapy to treat the psychological distress associated with EOC diagnosis [33]. While novel drugs were not identified, a filtered list of six was obtained for directed in vitro testing.

## Conclusions

Future research is needed to investigate the use of these CMAP–like analyses for determining combination therapies that might work synergistically to kill cancer cells and to apply this *in silico* bioinformatics approach using clinical outcomes to other cancer drug screening studies. Last of all, CMAP analyses only determines candidate drugs that can be tested in future studies with no information provided on the optimal dose in humans; research into the optimal therapeutic dosage needs to be considered in the planning of future drug studies [47].

## Additional files

Additional file 1: Figure S1. Results from clustering tumors from the TCGA and Mayo Clinic studies. Analyses were completed with genes found to be associated with clinical outcome in both studies (no adjustment for clinical covariates). (PNG 1066 kb)
Additional file 2: Figure S2. Results from clustering tumors from the TCGA and Mayo Clinic studies. Analyses were completed with genes found to be associated with clinical outcome in both studies (adjustment for clinical covariates). (PNG 1017 kb)
Additional file 3: Figure S3. The dose response data for 3-nitropropionic acid across the 10 EOC cell lines. (PNG 1781 kb)
Additional file 4: Figure S4,. The dose response data for 17-AAG across the 10 EOC cell lines. (PNG 1963 kb)
Additional file 5: Figure S5. The dose response data for adiphenine hydrochloride across the 10 EOC cell lines. (PNG 1763 kb)
Additional file 6: Figure S6. The dose response data for cephalexin across the 10 EOC cell lines. (PNG 1780 kb)
Additional file 7: Figure S7. The dose response data for clemizole across the 10 EOC cell lines. (PNG 1768 kb)
Additional file 8: Figure S8. The dose response data for cotinine across the 10 EOC cell lines. (PNG 1793 kb)
Additional file 9: Figure S9. The dose response data for doxorubicin across the 10 EOC cell lines. (PNG 2018 kb)
Additional file 10: Figure S10. The dose response data for ethosuximide across the 10 EOC cell lines. (PNG 1762 kb)
Additional file 11: Figure S11. The dose response data for mitoxantrone across the 10 EOC cell lines. (PNG 2056 kb)
Additional file 12: Figure S12. The dose response data for podophyllotoxin across the 10 EOC cell lines. (PNG 1927 kb)
Additional file 13: Figure S13. The dose response data for wortmannin across the 10 EOC cell lines. (PNG 1835 kb)
Additional file 14: Table S1. DGE and CMAP results. (XLSX 489 kb)

## Abbreviations

CMAP: Connectivity Mapping
DEGs: Differentially expressed genes
DMSO: Dimethyl sulfoxide
EOC: Epithelial ovarian cancer
HR: Hazard ratio
RPMM: Recursive partition mixture model
SEM: Standard error of the mean
TCGA: The Cancer Genome Atlas
TTR: Time to recurrence
