# Supplementary figures and images for: Drug discovery using clinical outcome-based Connectivity Mapping: application to ovarian cancer
Source: BMC Genomics. 2016 Oct 19;17:811. doi: 10.1186/s12864-016-3149-5 (PMC5069875; doi:10.1186/s12864-016-3149-5)

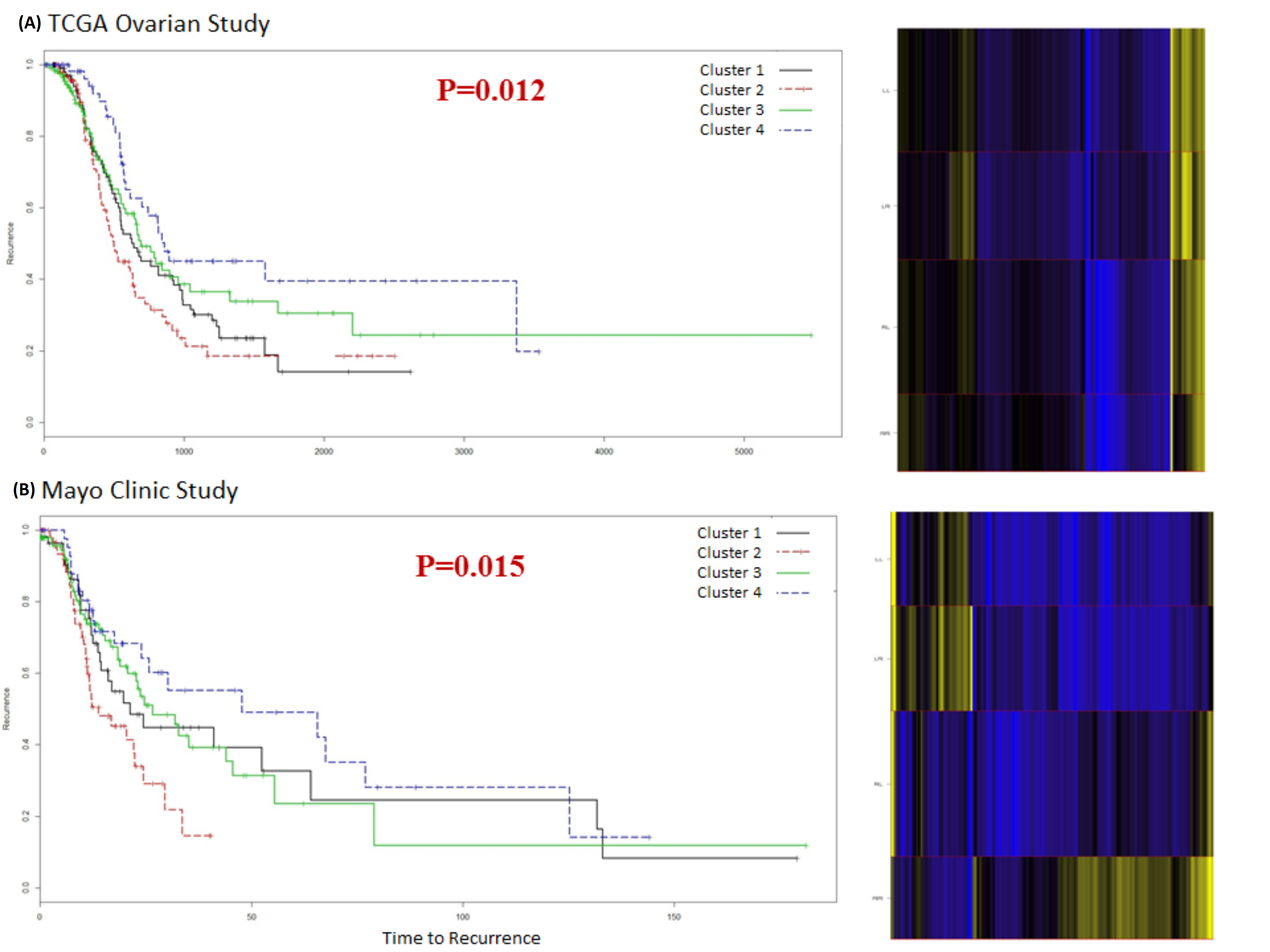

Supplement: Additional file 1: Figure S1. — Results from clustering tumors from the TCGA and Mayo Clinic studies. Analyses were completed with genes found to be associated with clinical outcome in both studies (no adjustment for clinical covariates). (PNG 1066 kb) [file 12864_2016_3149_MOESM1_ESM.png]

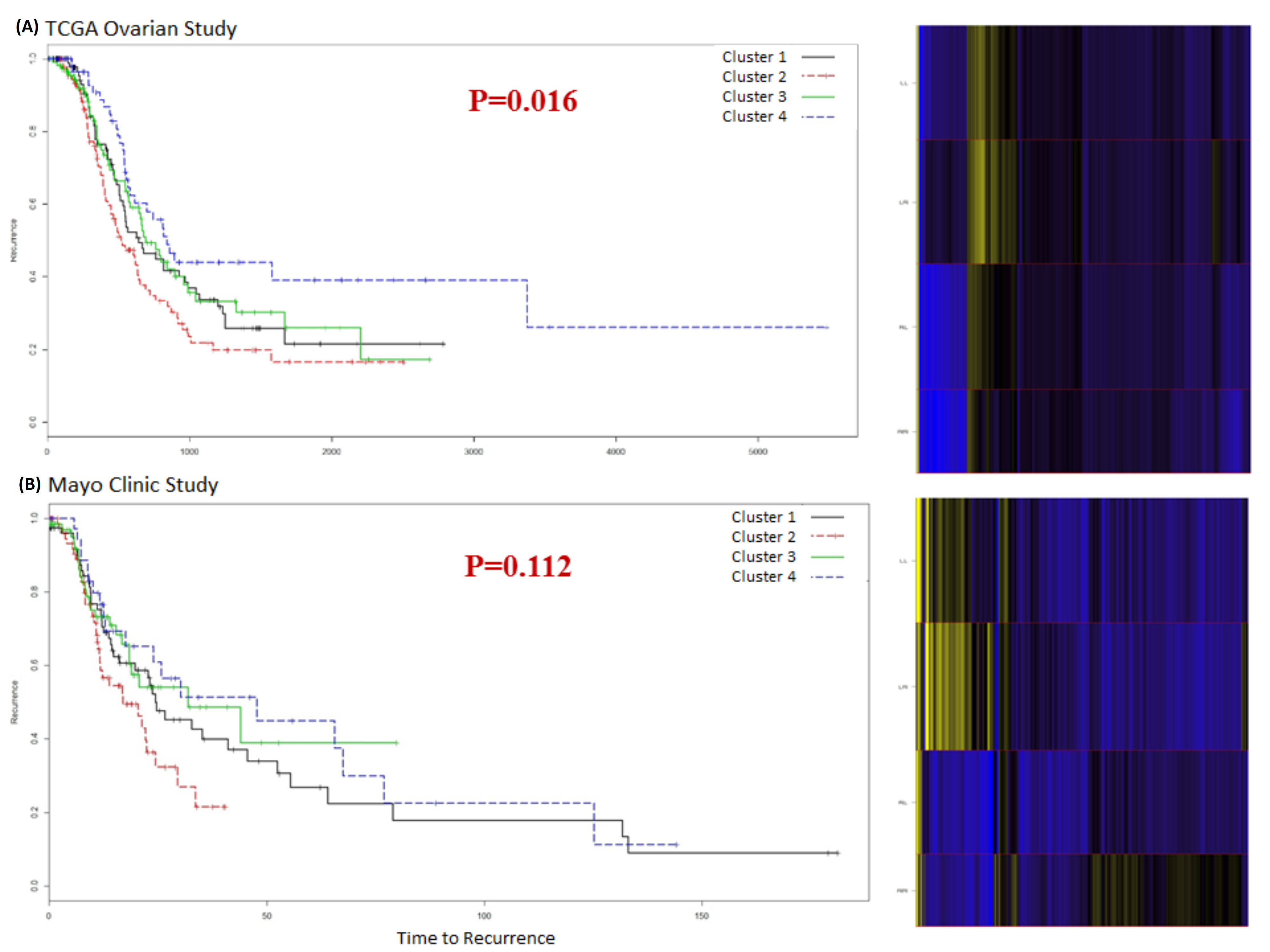

Supplement: Additional file 2: Figure S2. — Results from clustering tumors from the TCGA and Mayo Clinic studies. Analyses were completed with genes found to be associated with clinical outcome in both studies (adjustment for clinical covariates). (PNG 1017 kb) [file 12864_2016_3149_MOESM2_ESM.png]

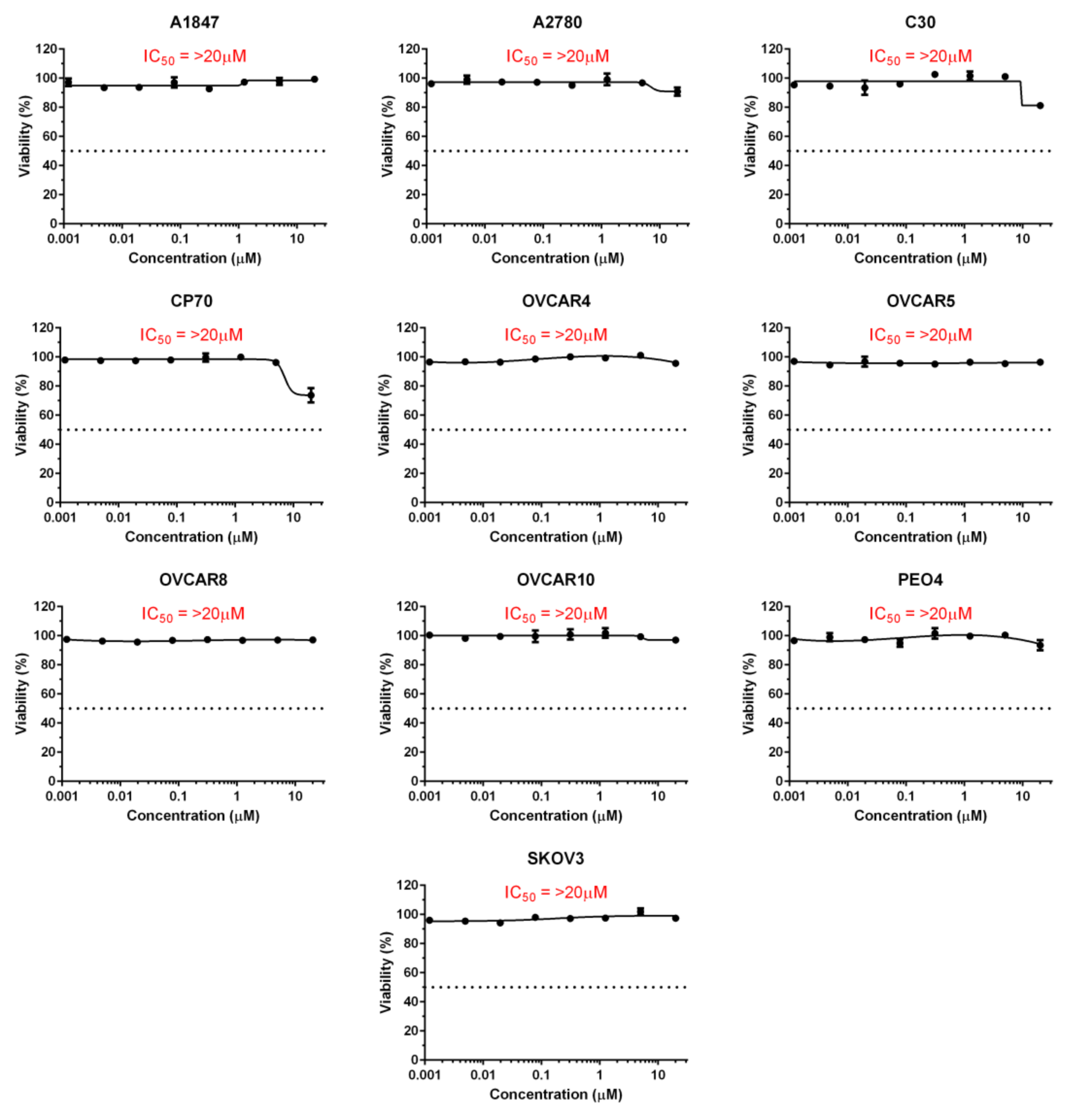

Supplement: Additional file 3: Figure S3. — The dose response data for 3-nitropropionic acid across the 10 EOC cell lines. (PNG 1781 kb) [file 12864_2016_3149_MOESM3_ESM.png]

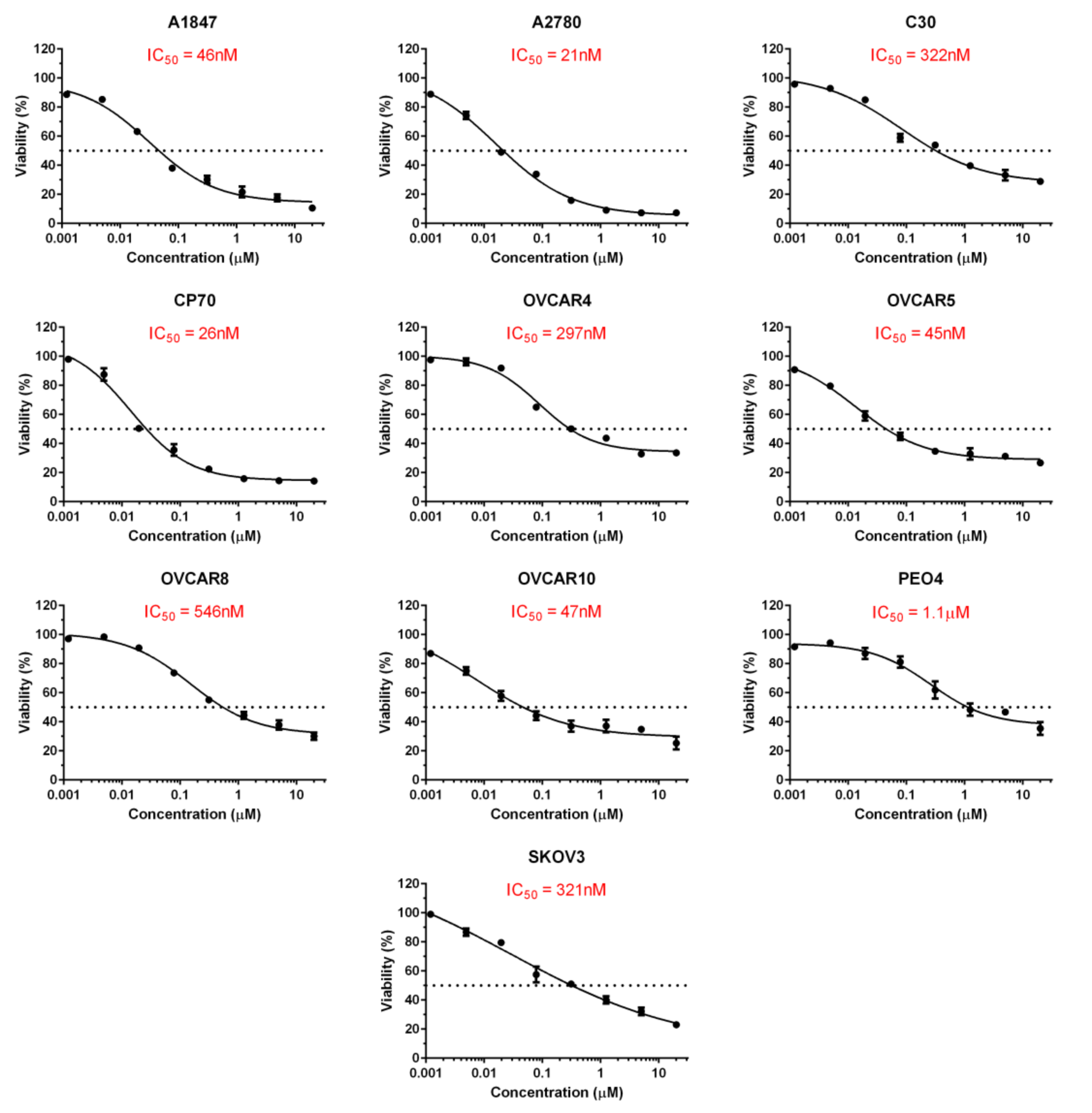

Supplement: Additional file 4: Figure S4,. — The dose response data for 17-AAG across the 10 EOC cell lines. (PNG 1963 kb) [file 12864_2016_3149_MOESM4_ESM.png]

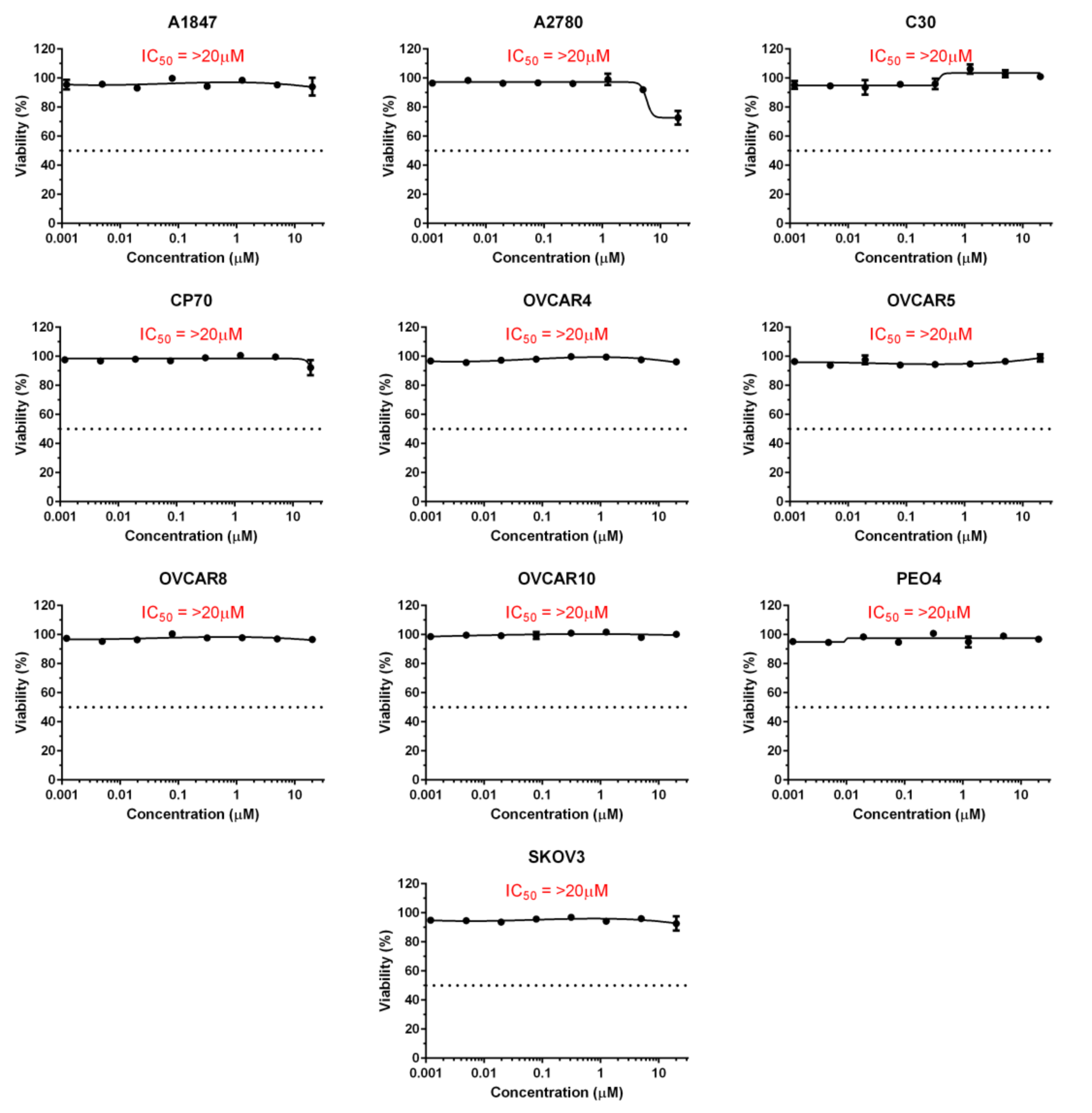

Supplement: Additional file 5: Figure S5. — The dose response data for adiphenine hydrochloride across the 10 EOC cell lines. (PNG 1763 kb) [file 12864_2016_3149_MOESM5_ESM.png]

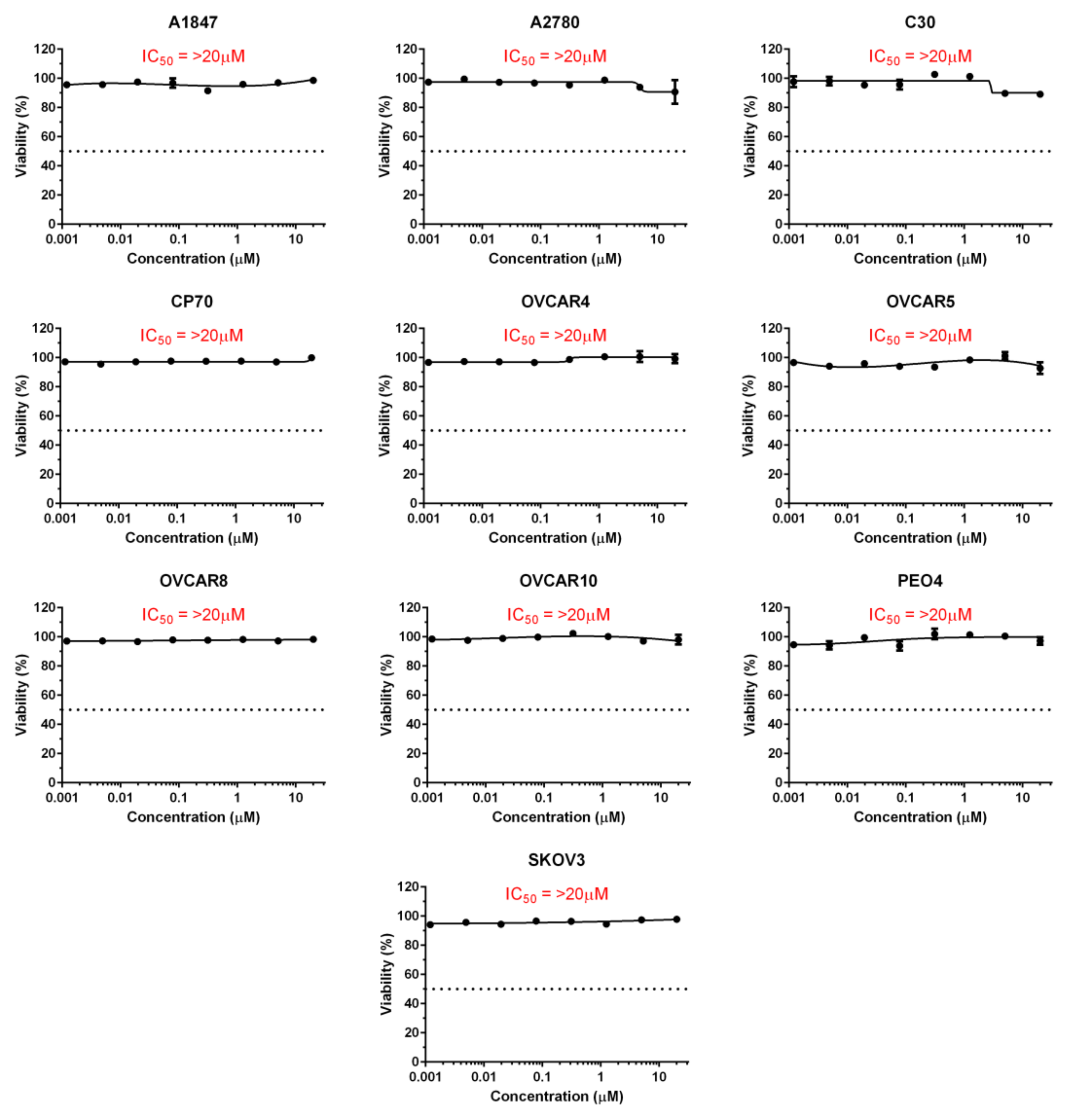

Supplement: Additional file 6: Figure S6. — The dose response data for cephalexin across the 10 EOC cell lines. (PNG 1780 kb) [file 12864_2016_3149_MOESM6_ESM.png]

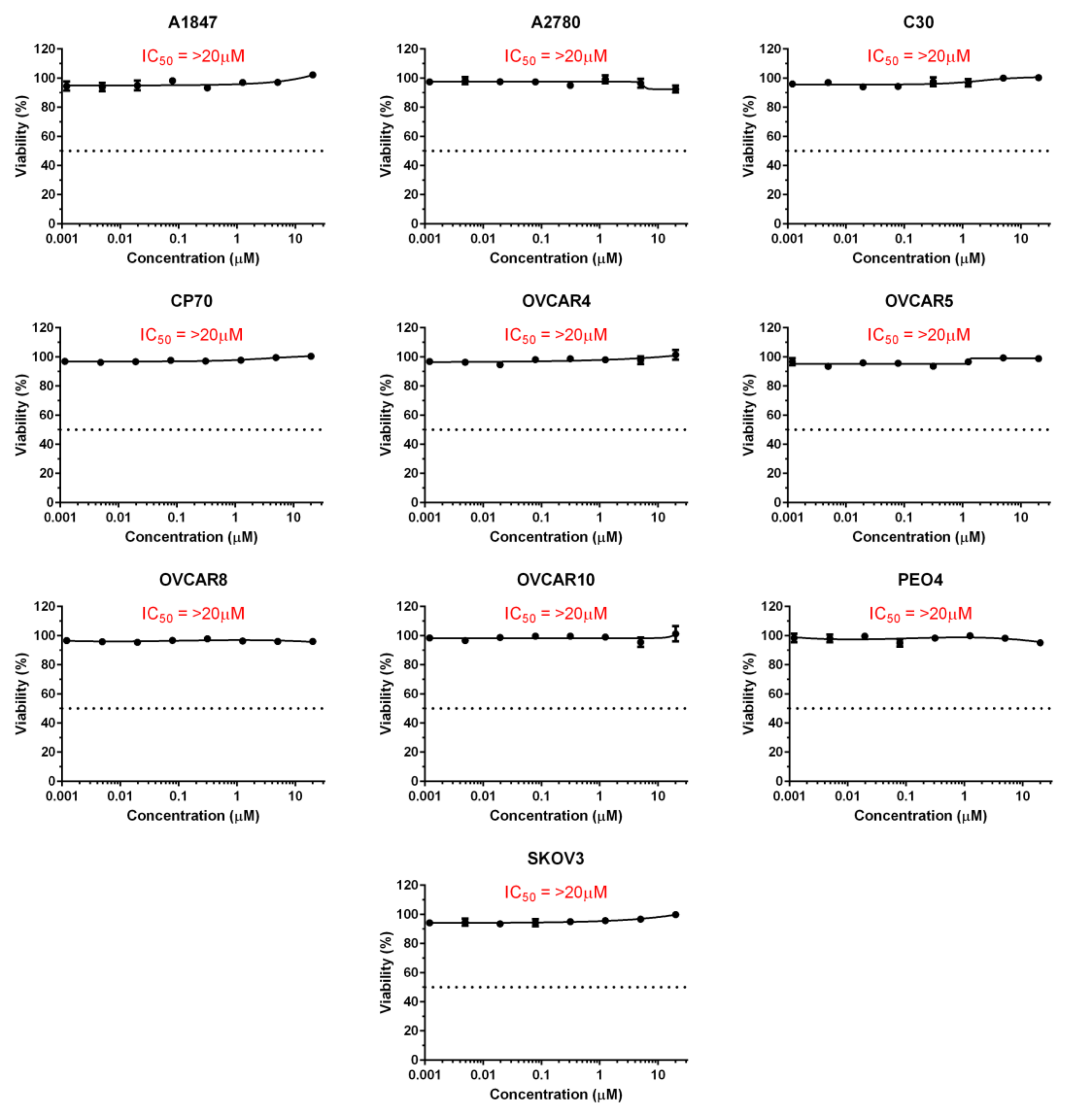

Supplement: Additional file 7: Figure S7. — The dose response data for clemizole across the 10 EOC cell lines. (PNG 1768 kb) [file 12864_2016_3149_MOESM7_ESM.png]

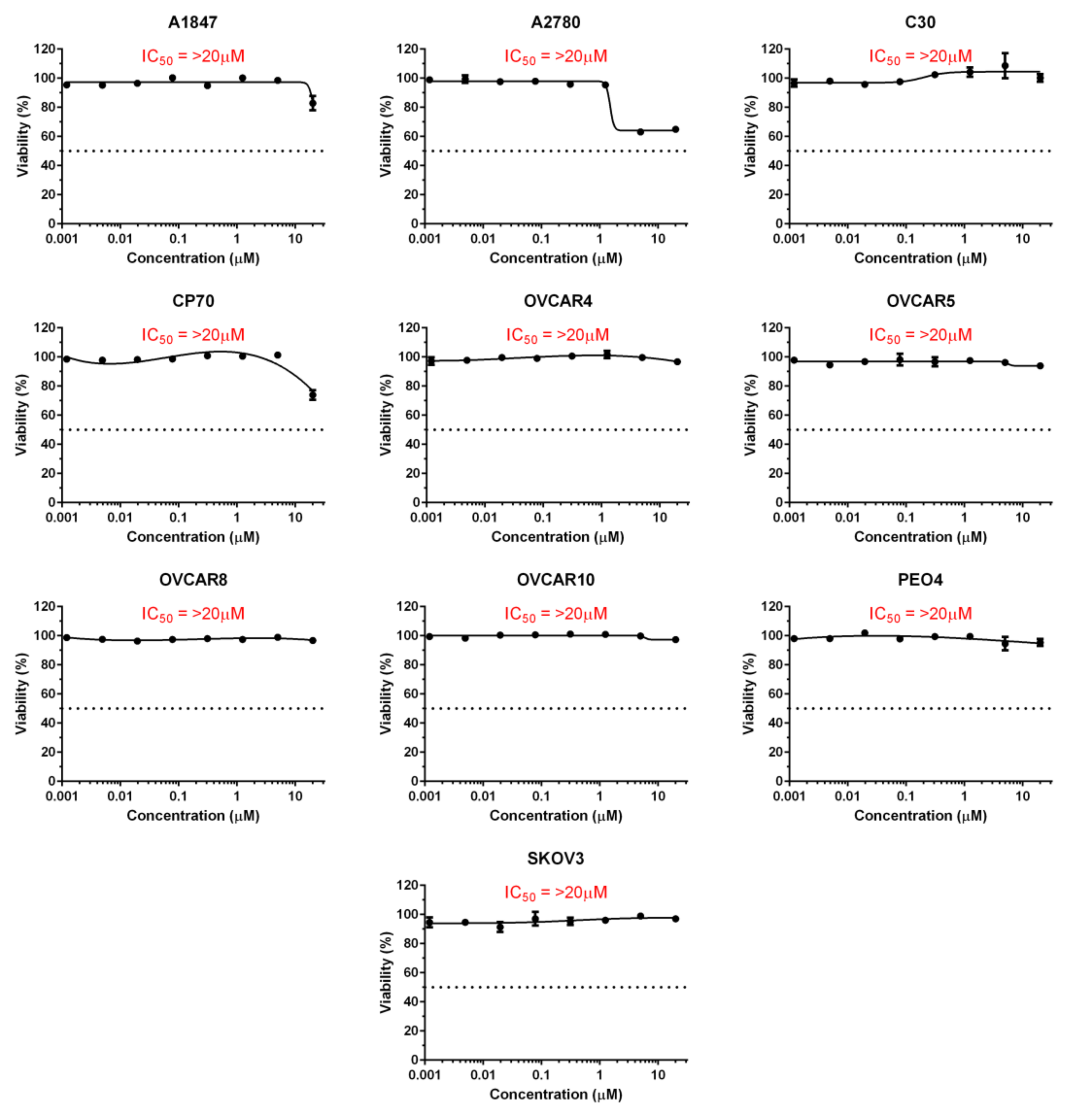

Supplement: Additional file 8: Figure S8. — The dose response data for cotinine across the 10 EOC cell lines. (PNG 1793 kb) [file 12864_2016_3149_MOESM8_ESM.png]

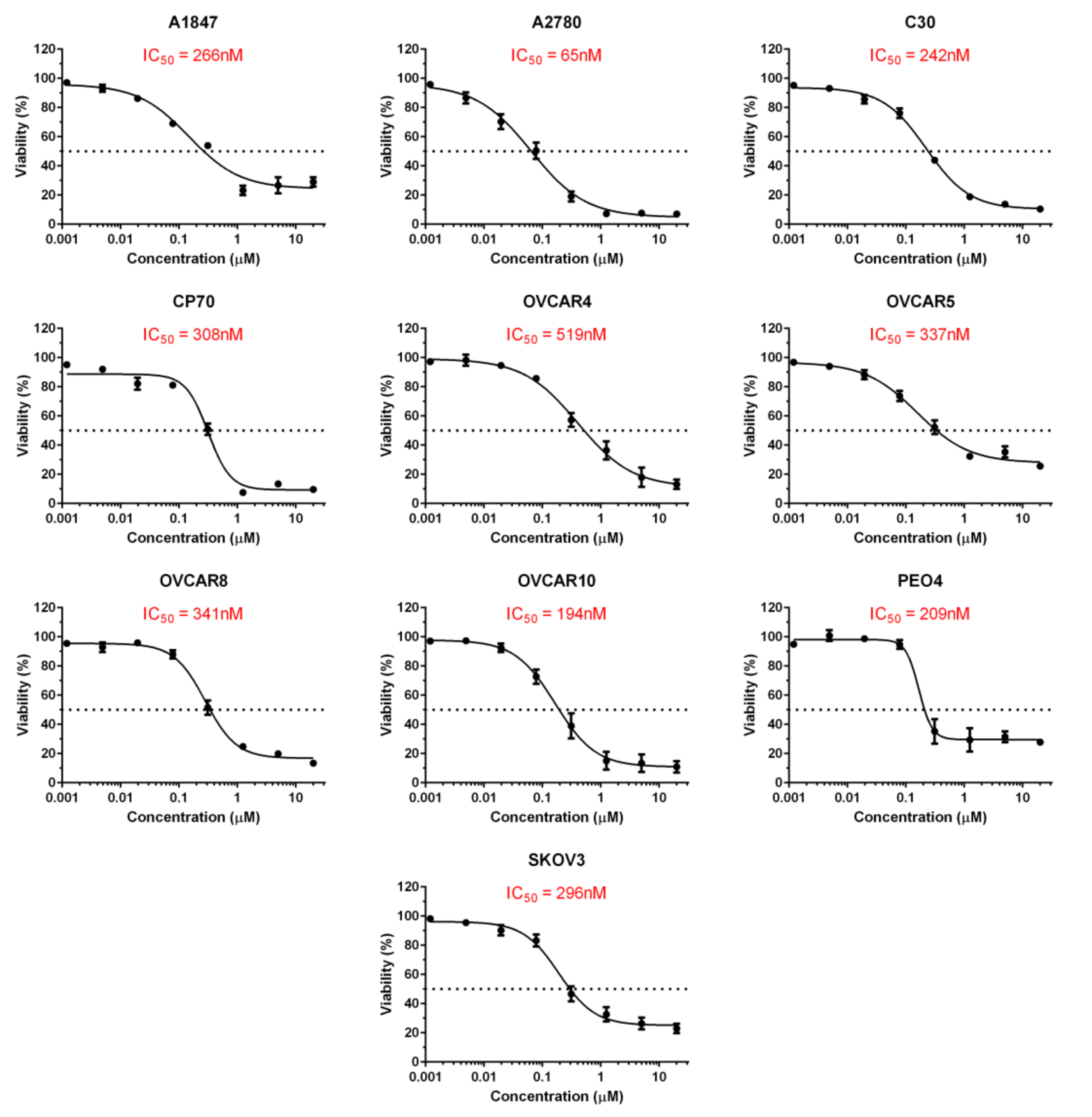

Supplement: Additional file 9: Figure S9. — The dose response data for doxorubicin across the 10 EOC cell lines. (PNG 2018 kb) [file 12864_2016_3149_MOESM9_ESM.png]

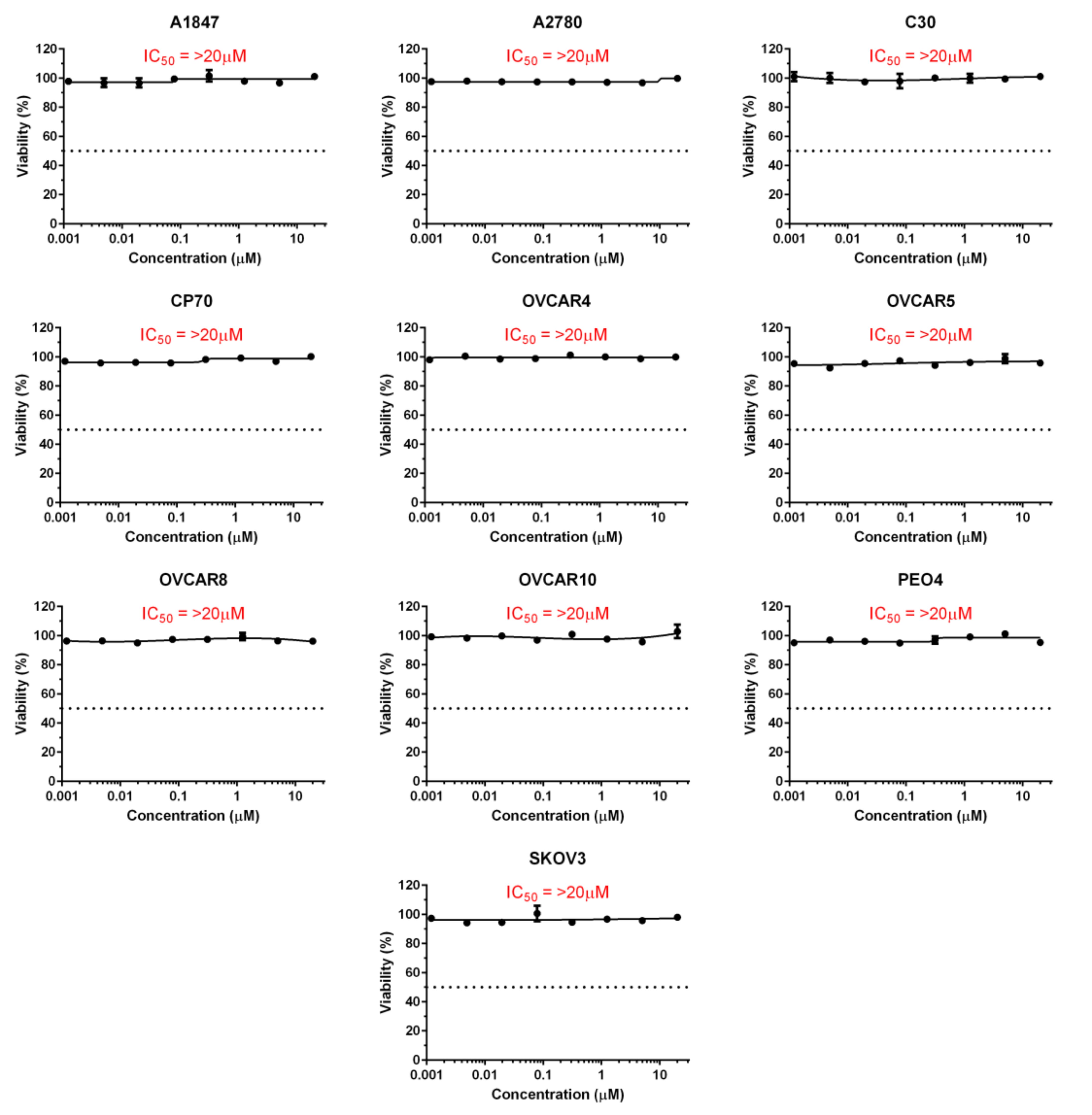

Supplement: Additional file 10: Figure S10. — The dose response data for ethosuximide across the 10 EOC cell lines. (PNG 1762 kb) [file 12864_2016_3149_MOESM10_ESM.png]

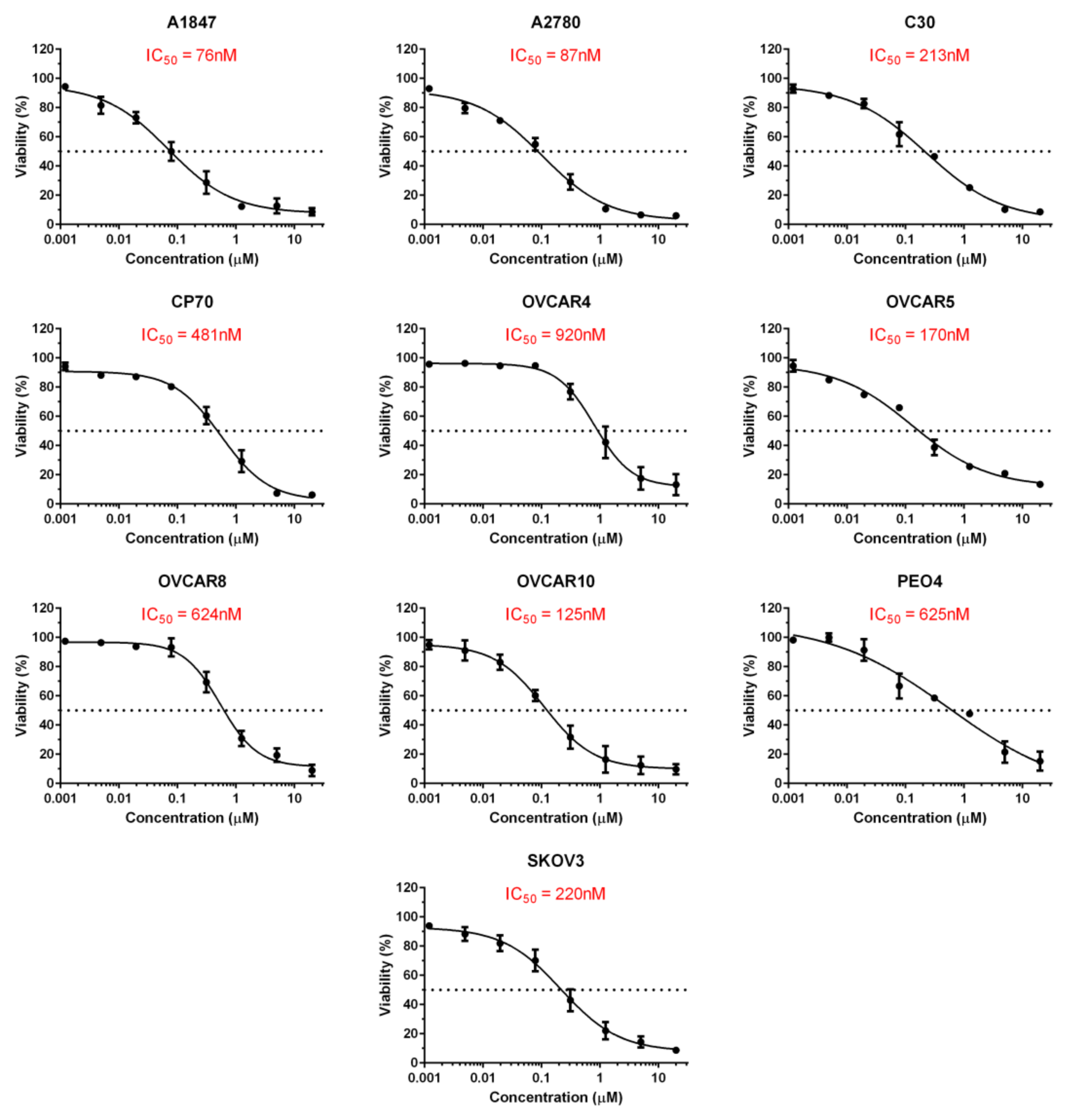

Supplement: Additional file 11: Figure S11. — The dose response data for mitoxantrone across the 10 EOC cell lines. (PNG 2056 kb) [file 12864_2016_3149_MOESM11_ESM.png]

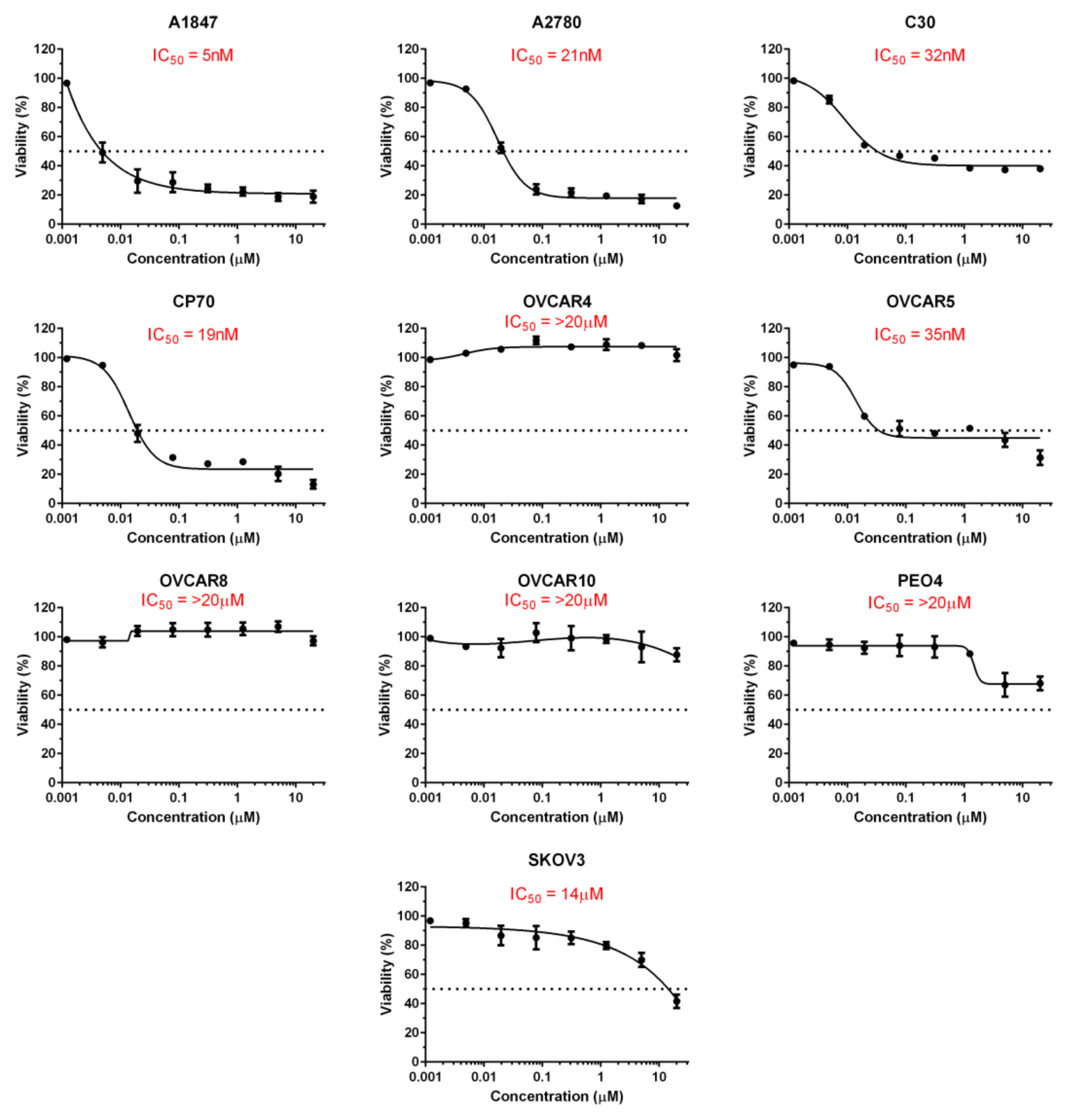

Supplement: Additional file 12: Figure S12. — The dose response data for podophyllotoxin across the 10 EOC cell lines. (PNG 1927 kb) [file 12864_2016_3149_MOESM12_ESM.png]

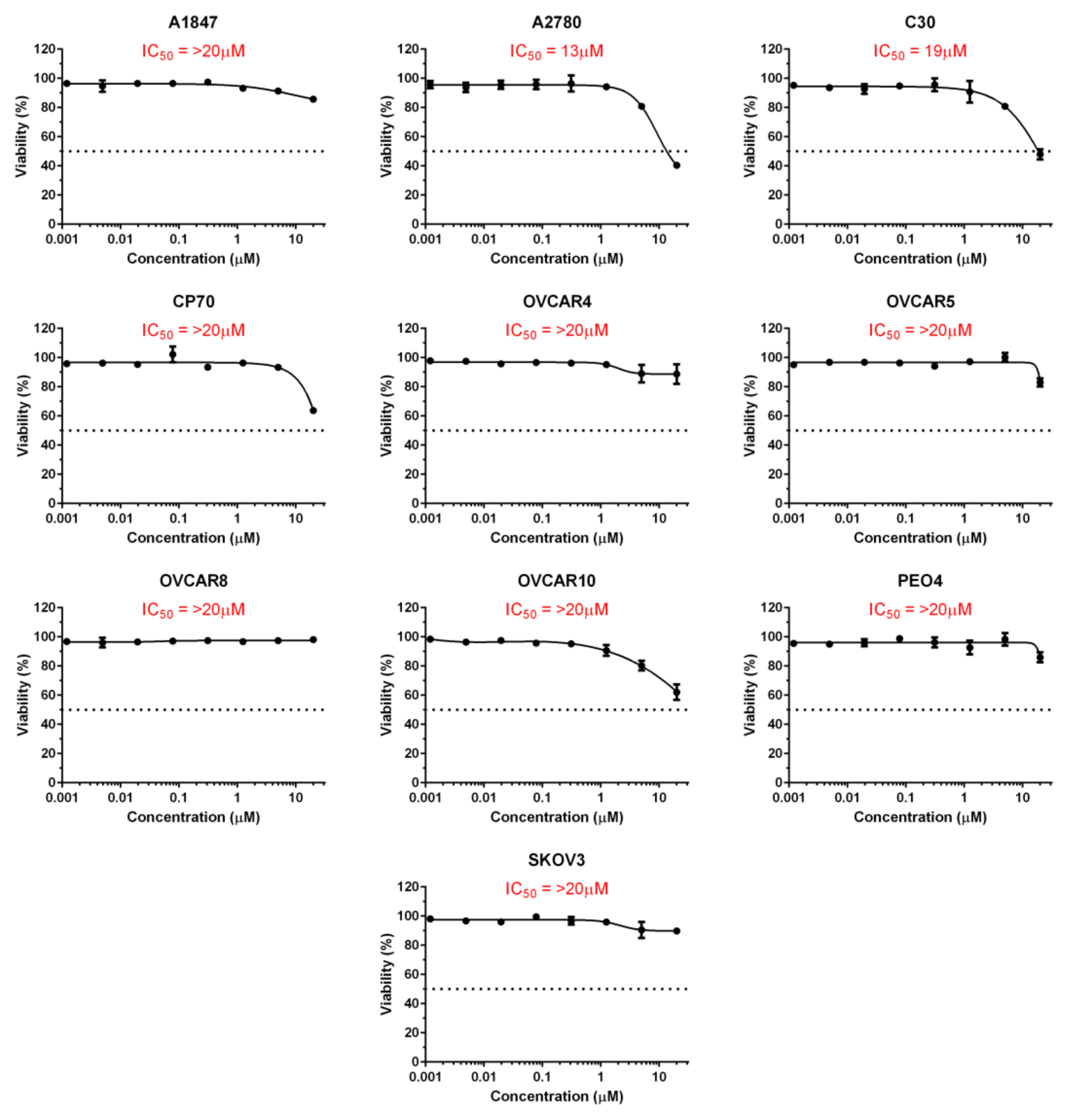

Supplement: Additional file 13: Figure S13. — The dose response data for wortmannin across the 10 EOC cell lines. (PNG 1835 kb) [file 12864_2016_3149_MOESM13_ESM.png]
